# Design of full-k-space flat bands in photonic crystals beyond the tight-binding picture

**DOI:** 10.1038/srep18181

**Published:** 2015-12-11

**Authors:** Changqing Xu, Gang Wang, Zhi Hong Hang, Jie Luo, C. T. Chan, Yun Lai

**Affiliations:** 1 College of Physics, Optoelectronics and Energy & Collaborative Innovation Center of Suzhou Nano Science and Technology, Soochow University, Suzhou 215006, China; 2 Department of Physics, Hong Kong University of Science and Technology, Clear Water Bay, Hong Kong

## Abstract

Based on a band engineering method, we propose a theoretical prescription to create a full-*k*-space flat band in dielectric photonic crystals covering the whole Brillouin Zone. With wave functions distributed in air instead of in the dielectrics, such a flat band represents a unique mechanism for achieving flat dispersions beyond the tight-binding picture, which can enormously reduce the requirement of permittivity contrast in the system. Finally, we propose and numerically demonstrate a unique application based on the full-*k*-space coverage of the flat band: ultra-sensitive detection of small scatterers.

In the past decades, a lot of attention has been focused on slow light and its applications such as optical information processing^1^,^2^,^3^,^4^,^5^,^6^,^7^,^8^,^9^. One possible route to realize slow light is the creation of flat bands in photonic crystals^2^,^3^,^4^,^5^,^6^,^7^,^8^,^9^,^10^,^11^,^12^,^13^,^14^,^15^, which exhibit dramatically small group velocities and high density of states (DOS), and thus provide an efficient method to slow down and control photons. In the typical band structures of photonic crystals, flat bands usually only appear in a small region in *k*-space, such as the Brillouin zone edge or center^16^,^17^,^18^,^19^,^20^,^21^,^22^,^23^. In order to expand the range of the flat bands so as to cover the whole Brillouin Zone, the conventional wisdom is to make the wave functions in the photonic crystal more localized in space. This can be easily understood within the framework of the tight-binding picture. When the wave functions on the sites are more localized, the coupling between neighboring sites becomes weaker, which in turn flattens the whole band. The tight-binding techniques for flat bands^24^ can be applied to coupled defect modes and coupled resonators^3^,^4^,^5^,^20^, or certain photonic crystals^25^,^26^, or coupled waveguides^27^,^28^. However, the wave scattering properties of photonic crystals only have the correspondence with quantum systems when 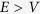, where 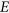 is the energy and 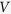 is the potential^29^, which is beyond the standard tight binding model. Therefore, new principles of flat band engineering beyond the tight-binding picture could exist in photonic crystals, as we shall demonstrate later.

In this work, we propose a theoretical prescription based on a band engineering method to create full-*k*-space flat bands covering the whole Brillouin Zone by designing the structure of photonic crystals. Comparing with normal flat bands with flatness only in one direction, such a full-*k*-space flat band can exhibit omnidirectional flatness, which leads to higher DOS and a unique application: ultra-sensitive detection of nearby small scatterers by enhancing the scattered waves in specific angles. In addition, the flat band provides resonances that are very sensitive to background permittivity, which is analogous to optical microcavities^30^,^31^. Surprisingly, the wave function distributions of some eigenstates in such a flat band are concentrated in air (free space) instead of dielectrics. This corresponds to wave functions localized between the sites instead of on the sites in a quantum system. Further analysis reveals that the wave functions exhibit quite small inverse participation ratios (IPRs), which indicate an unusual feature of delocalization in space. Since the permittivity is relatively low in our design, the flat band is potentially realizable in the infrared regime. By using full-wave simulations with a tiny gain, we have demonstrated ultra-sensitive detection of nearby small scatterers by such full-*k*-space flat bands, which can provide a convenient approach for detection and measuring of micro size particles.

We start with a typical two-dimensional square lattice of dielectric cylinders, as illustrated in the inset graph of Fig. 1(a). The radius and relative permittivity of the cylinders are set as 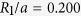 and 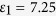, respectively, where 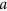 is the lattice constant. The calculated band structure for transverse electric (TE) polarization with the electric field parallel to the cylinders is plotted in Fig. 1(a). All results are plotted in terms of dimensionless frequency *2πc*/*a*, where *c* is the vacuum speed of light. It can be observed that the flat bands occur almost exclusively around the Γ, M, and X symmetry points in the Brillouin Zone. In order to expand the region of the flat band in *k*-space, we employ the band engineering method which was previously proposed to enlarge band gaps^32^,^33^. We first investigate four typical modes at the symmetry points Γ_3_, Γ_4_, M_4_ and X_5_ (the subscript marks the band index hereafter.) We found that Γ_3_, Γ_4_ and M_4_ modes are of almost the same frequency, while the X_5_ mode has a relatively higher frequency. The eigenfields of the Γ_3_, Γ_4_, M_4_ and X_5_ modes are shown in Fig. 1(b,e) respectively. For the Γ_3_ and Γ_4_ modes this structure exhibits doubly degenerate dipole modes with longitudinal and transverse polarizations, respectively. Interestingly, the M_4_ and X_5_ modes develop the pattern of field concentrating in air (free space) instead of dielectric cylinders. Especially, we note that eigenfields of both Γ_3_, Γ_4_ and M_4_ modes have zero amplitudes at the middles of the unit cell boundaries, while the eigenfield of X_5_ mode is nonzero there. Therefore, the frequency of the X_5_ mode can be shifted downwards by applying the band engineering method. When the permittivity distribution in the unit structure has a small perturbation from 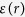 to 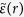, the eigenfrequency will be shifted from 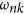 to 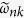 according to the following formula:


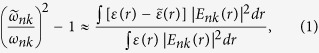


where 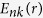 denotes the unperturbed modes. By applying this formula, it can be easily seen that by inserting small dielectric cylinders at the middles of the unit cell boundaries, it is possible to lower the frequency of the X_5_ mode, while keeping the frequencies of Γ_4_ and M_4_ modes almost unchanged. In this way, a full-*k*-space flat band over the whole Brillouin Zone can be created.

On the basis of the above analysis, two small dielectric cylinders with radius and relative permittivity set as 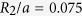 and 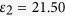, respectively, are added at the middles of the unit cell boundaries, as shown in the inset graph of Fig. 2(a). Figure 2(a) shows the new band structure for 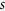 wave. It is found that an ultra-flat band (green circles) within 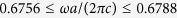 emerges above the first gap covering the whole Brillouin Zone. Comparing to the band structure without perturbations in Fig. 1, the frequencies of Γ_4_, Γ_5_ and M_4_ modes are almost not shifted, while the frequency of X_4_ mode is shifted downwards as desired, which flattens the band. To examine the flatness of the band in the whole *k*-space, we have plotted the fourth and fifth bands in the whole Brillouin Zone in Fig. 2(b). It is seen that the flat band is quite flat over the whole Brillouine Zone, i.e. the full *k*-space. The eigenfields of the Γ_4_, Γ_5_, M_4_ and X_4_ modes are shown in Fig. 2(c–f), respectively. From the eigenfields of Γ_4_ and Γ_5_ modes, it is seen that the flat band corresponds to a transverse band, while the band above the flat band is a longitudinal one. The eigenfields of the Γ_4_, Γ_5_ and M_4_ modes are almost the same as those in Fig. 1, while the eigenfield of the X_4_ mode is changed substantially, which leads to reduction of its frequency. Here, we emphasize that the eigenfields of the M_4_ and X_4_ modes are still concentrating in air. This indicates a significant difference from the flat bands created by enhancing permittivity which leads to wave localization in high permittivity. For a quantum correspondence, this corresponds to wave functions localized between the sites instead of on the sites and is beyond the tight-binding picture.

With the full coverage over the whole two-dimensional Brillouin Zone, the flat band exhibits an extraordinarily DOS beyond one-dimensional flat bands. Figure 3(a) displays the DOS of the photonic crystal, which exhibits a sharp peak at the frequency of the full-*k*-space flat band above a band gap. To characterize the feature of the eigenmodes belonging to the flat band, we calculate the inverse participation ratio (IPR) index^34^ in the unit cell defined by


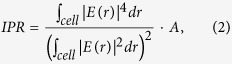


where 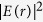 represents the intensity distribution of the electric field of eigenmodes and *A* is the area of the unit cell. It is worth noting that the integral is over the unit cell of the lattices, and thus the specified IPR represents a measure of the spatial distribution of the mode. For comparison, we have also calculated the IPR of the eigenmodes in a different flat band of similar flatness at a similar frequency, but created by high permittivity in a photonic crystal composed of rods with 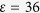 in a square lattice (see Supplementary Materials). Both the IPRs of the eigenfields in the two flat bands are shown in Fig. 3(b). It is clearly seen that IPR value of the engineered full-*k*-space flat band (green circles) is much lower than that of the high-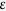-induced flat band (purple circles), This indicates a unique feature of spatial distribution of modes: the field distributed in free space instead of in dielectrics. In Fig. 3(c,d), we have plotted the equal frequency contour (EFC) and the absolute value of the group velocity (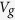) of the full-*k*-space flat band. Figure 3(c) shows that the maximum and minimum frequencies of this flat band are located in the ΓX and ΓM directions, respectively. Figure 3(d) shows that the maximum group velocity in the flat band is 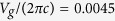 located between the maximum and minimum frequencies in the EFC. In Fig. 3(e), we demonstrate the calculated transmission spectrum of incident plane wave through a sample consisting of 10 layers of unit cells in the ΓX direction. The transmission spectrum shows a high transmittance regime sandwiched between two evanescently decaying regimes corresponding to the lower band gap and the higher longitudinal mode which cannot be excited by normal incidence^21^,^22^,^23^. The transverse nature of the flat band enables high coupling efficiency with external waves. Due to the extreme flatness of the band, we also observe extremely sharp Fabry-Perot resonances within such a small sample of only 10 layers of unit cells, less than 7 wavelengths in free space.

Here are some additional comments. We show that an ultra-flat band can be rationally designed using the perturbation theory. As a matter of fact, such a procedure can be iteratively applied to achieve a band as flat as we like if more complicated design is made in the unit structure of the photonic crystal. For example, by tuning the shapes and the parameters of cylinders in the aforementioned photonic crystal structure, we may asymptotically obtain a flat band with a vanishing group velocity (see Supplementary Materials).

With such dramatic coverage in k-space within a narrow frequency window, the flat band provides an excellent candidate for ultra-sensitive detection of small disturbance of energy and momentum of photons. In this work, we demonstrate two applications for ultra-sensitive detection of background permittivity and small scatterers. Firstly, such a flat band can be used for ultra-sensitive detection of background permittivity change. In Fig. 3(f), we show the dependence of the transmittance through the photonic crystal (as demonstrated in the inset graph) on the background permittivity 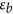. The frequency is chosen to be 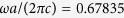, which corresponds to a transmission peak in the full-*k*-space flat band. It is seen that a tiny change of 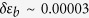 would dramatically reduce the transmittance from almost unity to less than 10%. This result is consistent with the sharp Fabry-Perot resonance peaks in the transmission spectra in Fig. 3(e).

Another interesting and unique application of such a full-*k*-space flat band is the ultra-sensitive detection of a small nearby scatterer. When electromagnetic waves are incident on the scatterers, scattered waves of other *k* components will be induced. For small particles, such scattered waves are too small to detect. However, when the scatterer is located nearby the photonic crystal with a full-*k*-space flat band, these small components in scattered waves with changed momentums could be selected out by the sharp Fabry-Perot effect in the flat band, and with a tiny gain, might even be enhanced to have comparable amplitudes of the incident waves. Such a mechanism is demonstrated in Fig. 4(a). Since the off-normal scattered wave components are enhanced instead of the normal component of incident wave, this application requires a full-*k*-space flat band rather than a one-dimensional one. To demonstrate this function, we have performed numerical simulations using COMSOL Multi-physics, as shown in Fig. 4(b). A tiny gain is added in the background of the photonic crystals as 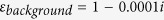. The thickness of the sample is about 8 wavelengths in free space. The upper and lower boundaries are set as Floquet periodic conditions. The working frequency is chosen to be *ωa*/(2*πc*) = 0.6766. When a tiny light scatterer of 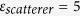 is placed in front of the photonic crystals, the electric field distributions inside the photonic crystal and on the other side are both changed dramatically, as demonstrated in Fig. 4(b). To analyze the far-field transmitted waves, we plot the angular distribution of far-field transmission in Fig. 4(c), which is obtained by Fast Fourier transform of the transmitted field at the dot line 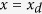 in Fig. 4(b), i.e.


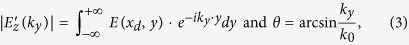


where 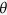 is the angle of transmission and 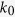 is the eigenvector. It is seen that when there is no scatterer, there is only one transmission peak at 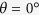. When a small scatterer of radius 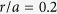 is placed beside the photonic crystal, three pairs of off-normal peaks arise at 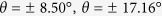 and 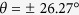. When the small gain is added in the background, the amplitude of the transmission is further enhanced greatly, even to the scale of the incident field (which is unity). Therefore, through analyzing the far-field radiation or the field distributions inside the photonic crystals, small scatterers near the sample can be detected efficiently. This unique function is enabled by the large DOS of the omnidirectional flat band. In this system, the largest permittivity (21.5) is within the permittivity range in the infrared regime^35^ and can be realized in experiment^36^. In principle, such a full-*k*-space flat band system can be realized at infrared frequencies and thus enables the ultra-sensitive detection of micrometer scatterers.

Our flat band system can also be applied in slow light waveguides. We note that full-*k*-space flat band is not a necessary condition for slow-light waveguide systems. Since the two-dimensional full-*k*-space flat band system is above the light line in vacuum, it may possess a high radiation loss. Therefore, for slow light applications, our system should be sandwiched by two mirrors that limit the radiation loss.

In summary, we report the creation of nearly flat bands over the whole Brillouin Zone, i.e. full-*k*-space flat bands, by applying the band engineering method in photonic crystals. Interestingly, eigenstates in such flat bands can exhibit disperse wave functions that are distributed in air instead of in the dielectrics. Consequently, flat dispersions can be achieved with smaller permittivity contrast. Based on the full-*k*-space coverage of the flat band, a unique application for ultra-sensitive detection of nearby small scatterers is proposed and demonstrated. Our work represents a general principle of flat band engineering in classical wave systems beyond the tight binding picture.

## Additional Information

**How to cite this article**: Xu, C. *et al.* Design of full-k-space flat bands in photonic crystals beyond the tight-binding picture. *Sci. Rep.* **5**, 18181; doi: 10.1038/srep18181 (2015).

## Supplementary Material

Supplementary Information

## Figures and Tables

**Figure 1:**
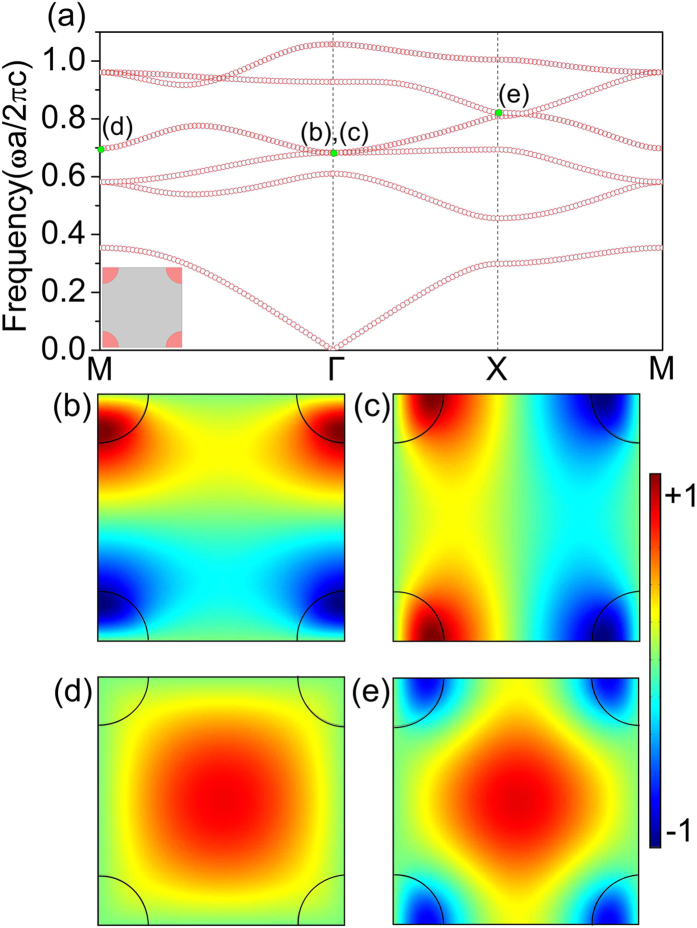
Band structure and eigenfields of a typical two-dimensional square lattice of dielectric cylinders. (**a**) Photonic band structure for the TE polarization (with the electric field parallel to the cylinders) in a 2D photonic crystal constructed by a square lattice of dielectric cylinders with radius and relative permittivity being *R*_1_/*a* = 0.200 and *ε*_1_ = 7.25, (*a* is the lattice constant). Eigenfields of (**b**) Γ_3_, (c) Γ_4_, (d) M_4_ and (e) X_5_ modes are shown. The inset to (a) is the pictorial representation of the unit cell.

**Figure 2:**
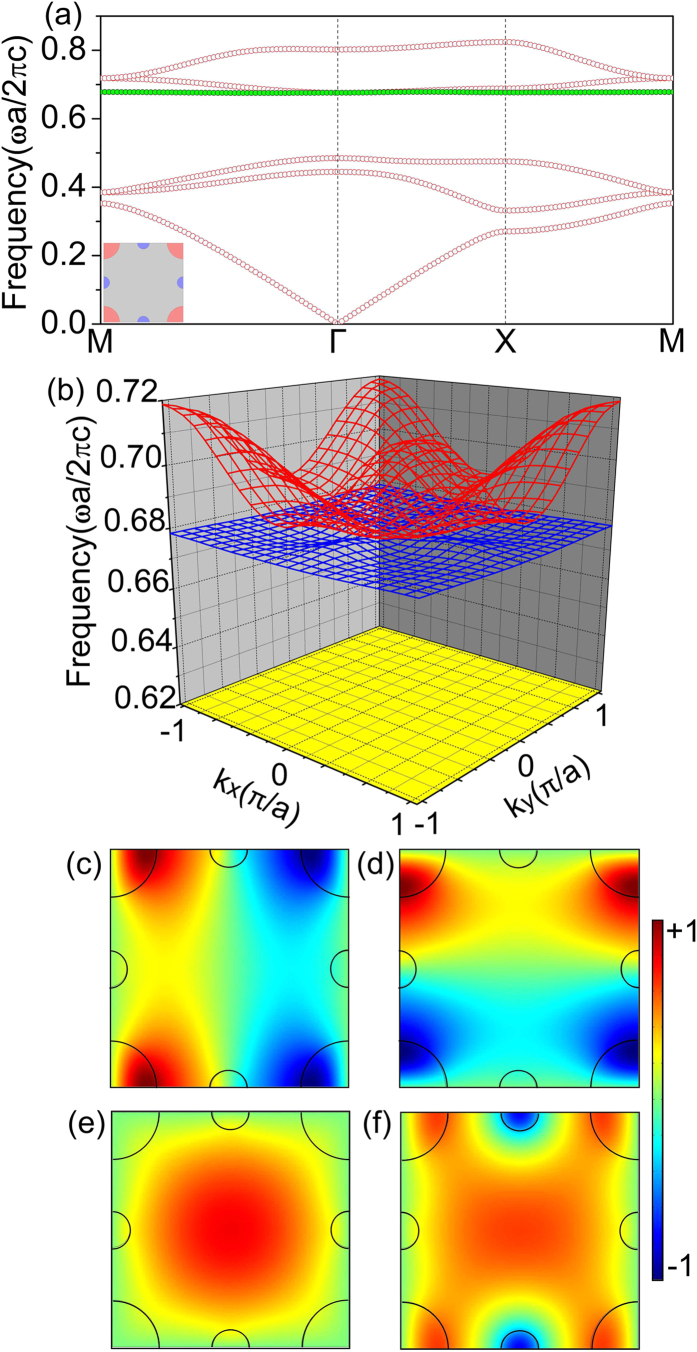
New band structure and eigenfields of the engineered structure with a full-*k*-space flat band. (**a**) The new band structure with a full-*k*-space flat band (green circles). (**b**) The fourth and fifth bands in the whole Brillouin zone. Eigenfields of the full-*k*-space flat band at (**c**) Γ_4_, (**d**) Γ_5_, (**e**) M_4_ and (**f**) X_4_ modes are plotted.

**Figure 3:**
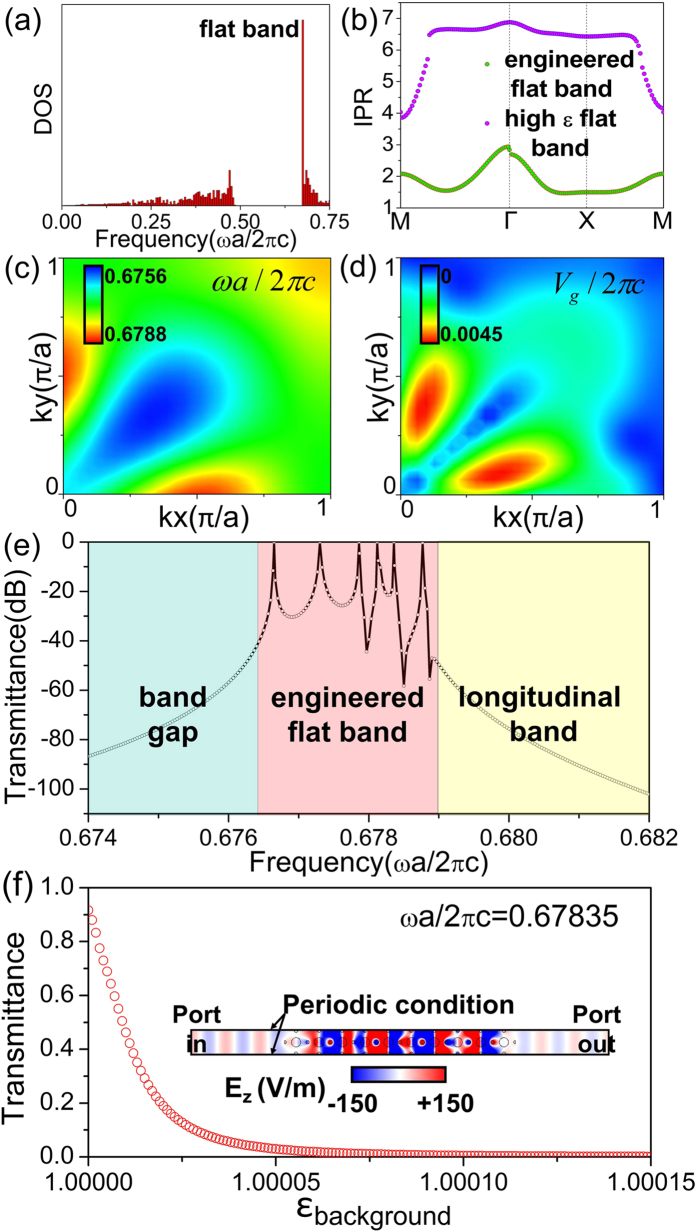
The characteristics of the full-*k*-space flat band. (**a**) The DOS of the photonic crystal with a sharp peak at the frequency of the engineered full-*k*-space flat band. (**b**) The IPR of the engineered flat band and a high-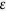-induced flat band. (**c**) The EFC of the engineered flat band. (**d**) The absolute value of group velocity (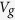) of the engineered flat band. (**e**) The calculated transmission spectrum near the engineered flat band in the ΓX direction. (**f**) The dependence of the transmittance on background permittivity at *ωa*/(2*π*c) = 0.67835.

**Figure 4:**
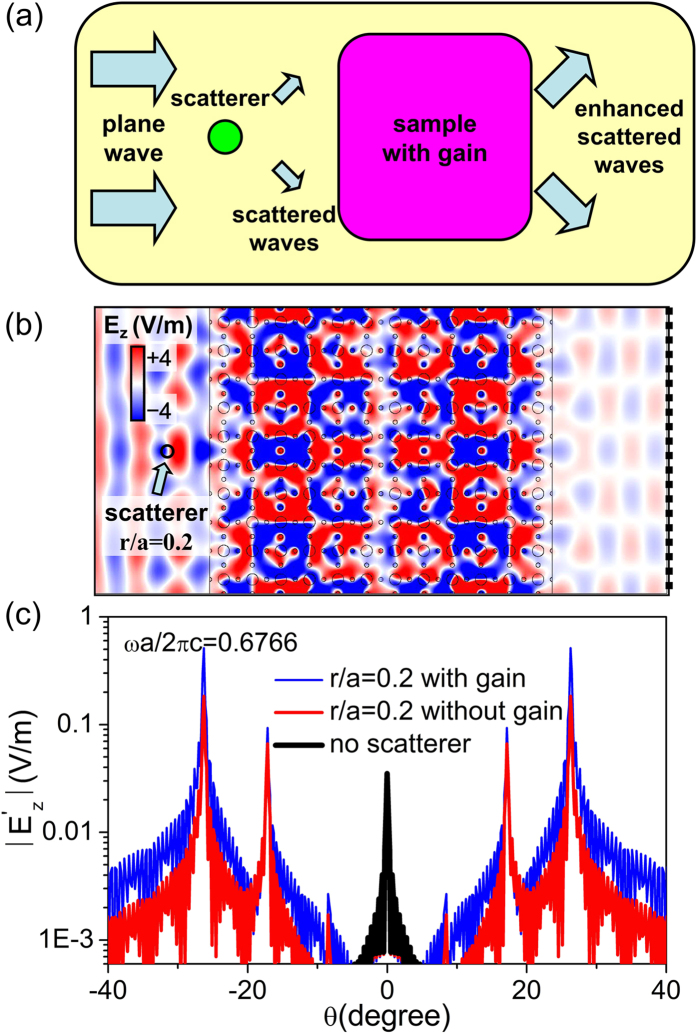
A unique application based on the full-*k*-space coverage of the flat band. (**a**) When a small scatterer is close to the photonic crystal with a full-*k*-space flat band, some components in scattered waves could be picked out and be enhanced. (**b**) The simulated field patterns at the frequency *ωa*/(2*π*c) = 0.6766 are plotted when a cylinder scatterer with *ε*_*scatterer*_ = 5 and 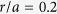 is placed near the sample. (**c**) Fast Fourier transform of the transmission field at dot line 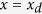 in (b) is shown.
