# Supplementary material for: Design of full-k-space flat bands in photonic crystals beyond the tight-binding picture
Source: Sci Rep. 2015 Dec 11;5:18181. doi: 10.1038/srep18181 (PMC4676056; doi:10.1038/srep18181)
Supplement: Supplementary Information [file srep18181-s1.doc]

Supplementary Information of “Design of full-k-space flat bands in photonic crystals beyond the tight-binding picture”

Changqing Xu^1^, Gang Wang^1^, Zhi Hong Hang^1^, Jie Luo^1^, C.T. Chan^2^, Yun Lai^1^*

^1^College of Physics, Optoelectronics and Energy & Collaborative Innovation Center of Suzhou Nano Science and Technology, Soochow University, Suzhou 215006, China

^2^Department of Physics, Hong Kong University of Science and Technology, Clear Water Bay, Hong Kong

Emails:[laiyun@suda.edu.cn](mailto:laiyun@suda.edu.cn)

**1. The engineering process of the flat band.**

Here, we show the engineering process of the full-k-space transverse flat band by the band engineering method. In Fig. S1, the parameters of cylinder 1 and the radius of cylinder 2 are the same as those in Fig. 2. The relative permittivity of cylinder 2 $\left(\varepsilon_{2}\right)$ is gradually enhanced from 1 to 30. During this process, certain modes (such as Fig. 2(f)) are shifted downward, while other transverse modes in the flat band are almost not shifted. The fourth band (painted green) undergoes a flattening process for $1\leq\varepsilon_{2}\leq21.5$ and an un-flattening process for $\varepsilon_{2}>21.5$.


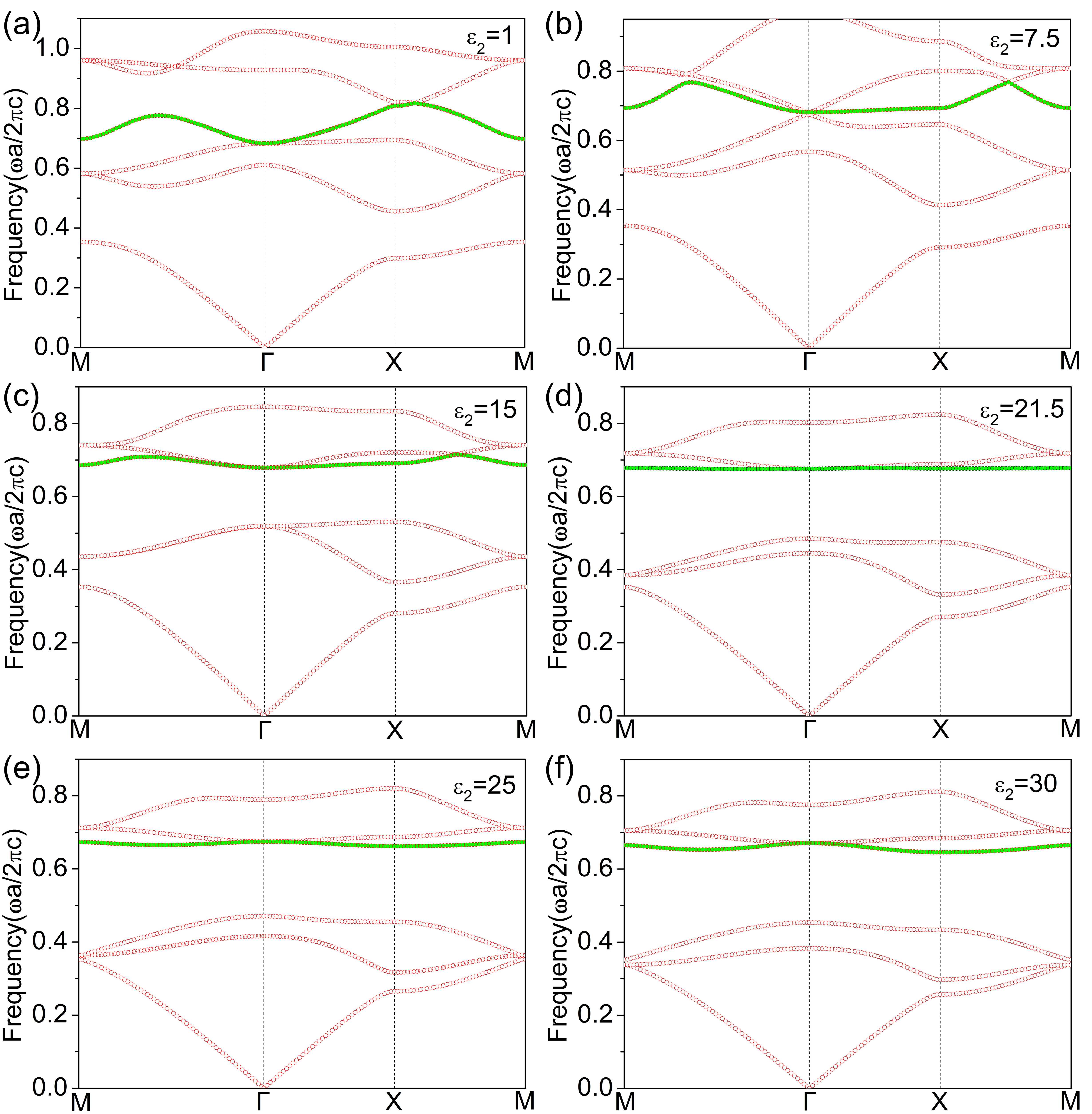


**Fig. S1 |** **The gradual change of band structure when $\varepsilon_{2}$ changes.** (a) $\varepsilon_{2}=1$, (b) $\varepsilon_{2}=7.5$, (c) $\varepsilon_{2}=15$, (d) $\varepsilon_{2}=21.5$, (e) $\varepsilon_{2}=25$, and (f) $\varepsilon_{2}=30$.

With the same lattice structure as that in Fig. 1, Fig. S2 shows the eigenfields of the $A_{4}$ (near the midpoint of $\Gamma M$ direction) and $\Gamma_{5}$ modes. Both of them have nonzero amplitudes at the middles of the unit cell boundaries. Therefore, by inserting small cylinders at the middles of the unit cell boundaries, their eigen-frequencies can be shifted downwards in the engineering process. $A_{4}$ is shifted downwards such that the flat band shown in the main text is formed. $\Gamma_{5}$ is shifted downwards more such that its frequency becomes even lower than the original $\Gamma_{3}$ and $\Gamma_{4}$ degenerate modes and opens the band gap below the flat band.


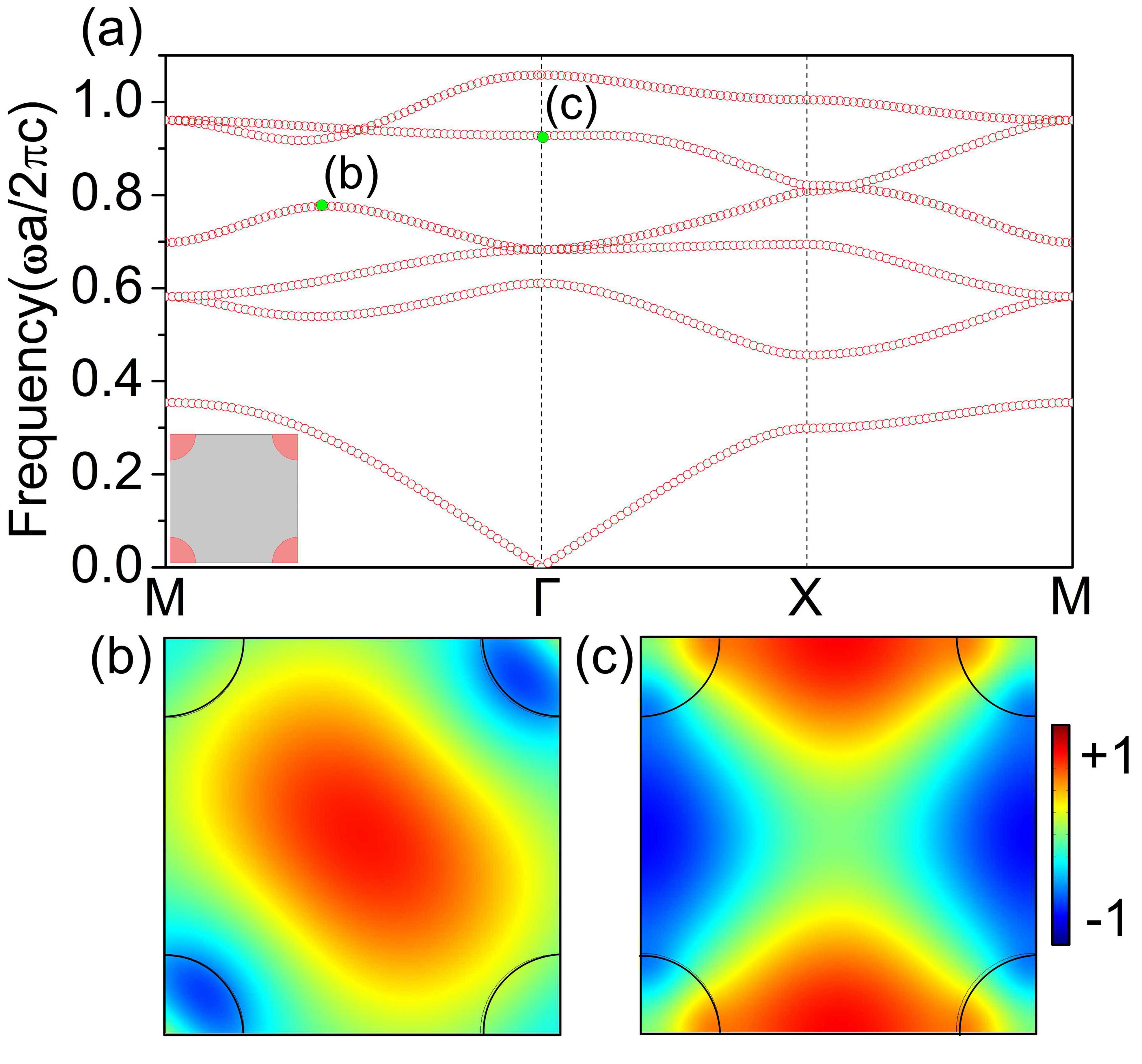


**Fig. S2** **|** (a) Photonic band structure with the same lattice structure in Fig. 1. Eigenfields of the (b) $A_{4}$ (near the midpoint of $\Gamma M$ direction) and (c) $\Gamma_{5}$ modes are shown.

**2. A high-$\varepsilon$-induced flat band with similar flatness to that in Fig. 2 at a similar frequency.**

Fig. S3 shows the calculated band structure with a photonic crystal composed of rods with high $\varepsilon=36$ in a square lattice. A flat band (painted green) with similar flatness to that in Fig. 2 at a similar frequency is formed by localized modes inside the high $\varepsilon$ rods. The localized modes have angular momentum quantum number $m=3$. The eigenfield of the $\Gamma$ point in the flat band is illustrated in the inset graph.


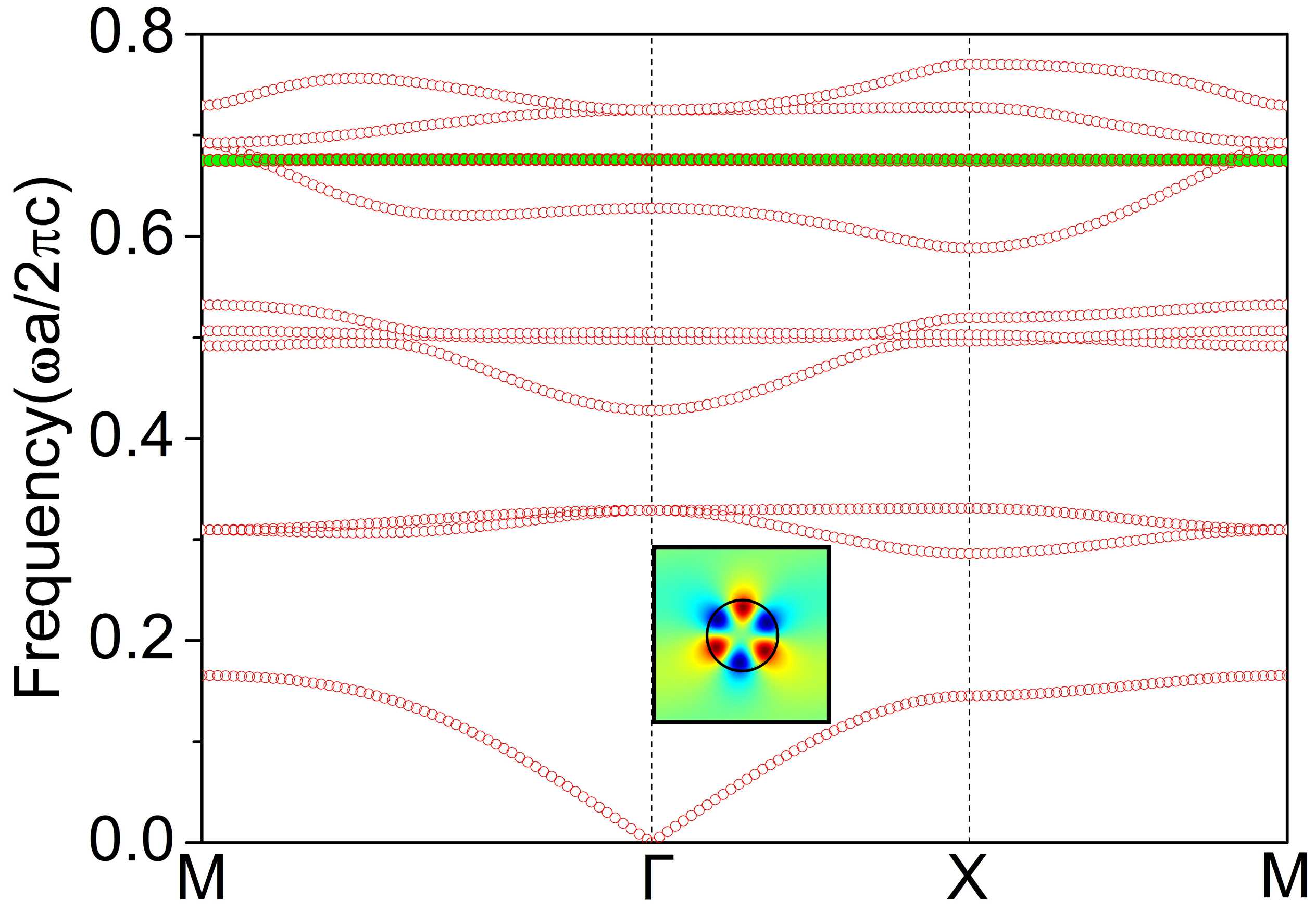


**Fig. S3 | The calculated band structure which exhibits a flat band (painted green) of similar flatness to that in Fig. 2 at a similar frequency.**

Such a flat band represents the normal method to create flat band: increasing the localization of wave functions on sites such that the interaction between sites are weaker. The high $\varepsilon$ of rods and larger angular momentum of modes leads to more localized wave functions in the rods. This method is in accordance with the tight-binding model.

**3. The further flattened band and further reduced group velocity.**

As illustrated in Fig. S4, we consider a two-dimensional structure which is similar to the structure in Fig. 2. The cylinder 1 with radius $R_{1}/a=0.20$ and relative permittivity $\varepsilon_{1}=7.2$ is located on the corner of the unit cell. The dielectric cylinder 2 in Fig. 2 is replaced by an elliptic cylinder 2 with relative permittivity $\varepsilon_{2}=30$. The semi-major axis (which is perpendicular to the boundary of unit cell) and the semi-minor axis of the elliptic cylinder 2 are set as $A/a=0.075$ and $B/a=0.040$. With the eigenfields similar to Fig. 2, the elliptic dielectric cylinders affect $\Gamma_{4}$ and $M_{4}$ modes more slightly but affect $X_{5}$ mode more intensely. As a result, more flat band can be achieved. The band structure, the EFC and the absolute value of the group velocity are plotted in Fig. S4(a), Fig. S4(b) and Fig. S4(c) respectively. The frequency range of the flat band is $0.6809\leq\omega a/2\pi c\leq0.6838$; the maximum group velocity in the flat band is $V_{g}/\left(2\pi c\right)=0.0038$.


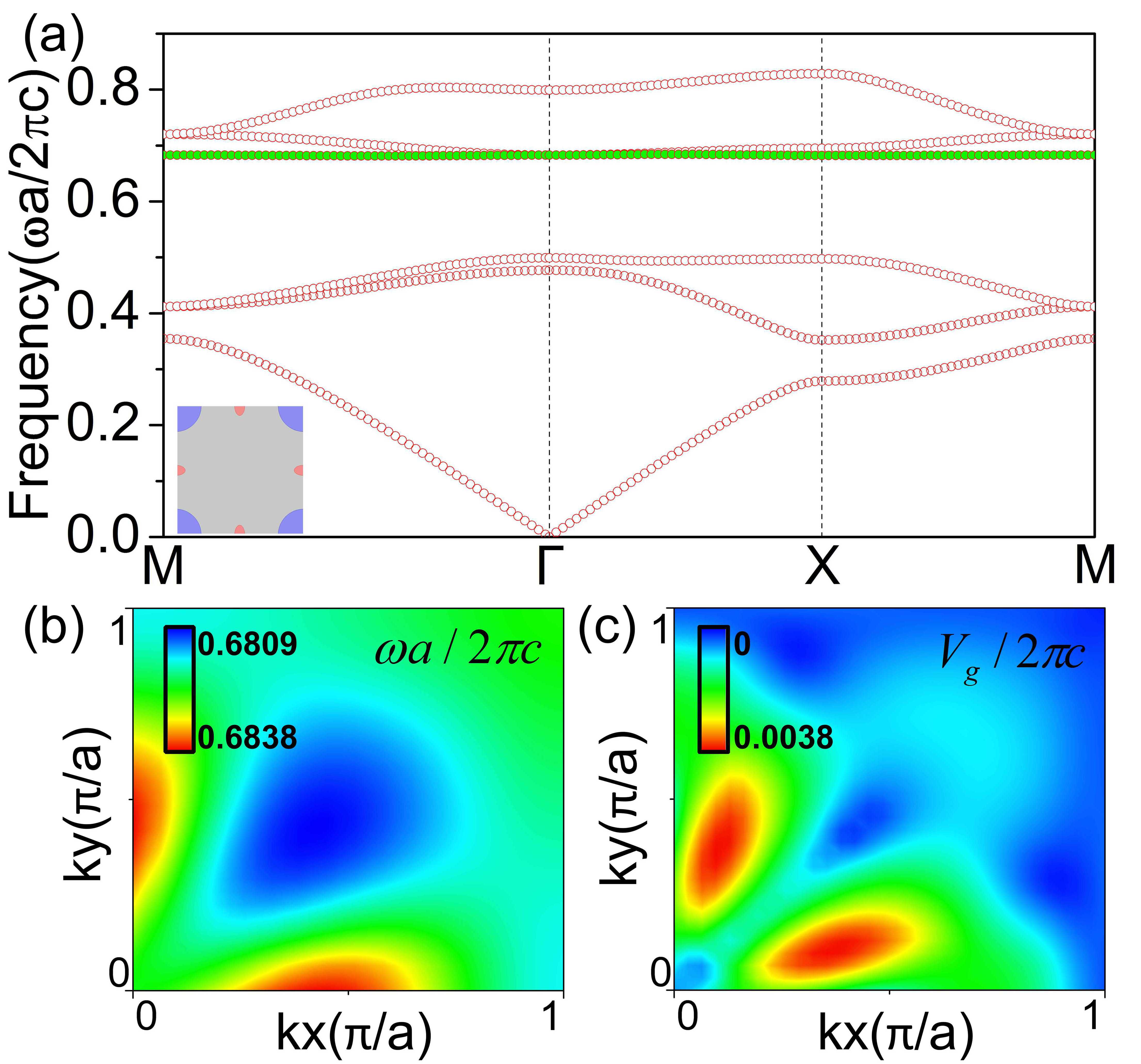


**Fig. S4 | The cylinder 2 in Fig. 2 is replaced by an elliptic cylinder.** (a) The band structure, (b) The EFC, (c) The absolute value of group velocity of the flat band is plotted.

If we are willing to attain a more flat band, an additional cylinder 3 at the center of unit cell is sufficient. In this case, the eigenfield for the $M_{4}$ mode is more concentrated on the cylinder 3. Furthermore, the cylinder 2 in Fig. 2 is set to be tangent to the boundary of unit cell. It is possible to adjust the frequencies of $M_{4}$, $\Gamma_{4}$ and $X_{4}$ modes independently. When the radius of cylinders are set as $R_{1}/a=0.20$, $R_{2}/a=0.05$ and $R_{3}/a=0.083$, the relative permittivity are set as $\varepsilon_{1}=15.0$, $\varepsilon_{2}=47.0$ and $\varepsilon_{3}=7.6$, the flat band is further flattened. The band structure, the EFC and the absolute value of the group velocity are plotted in Fig. S5.


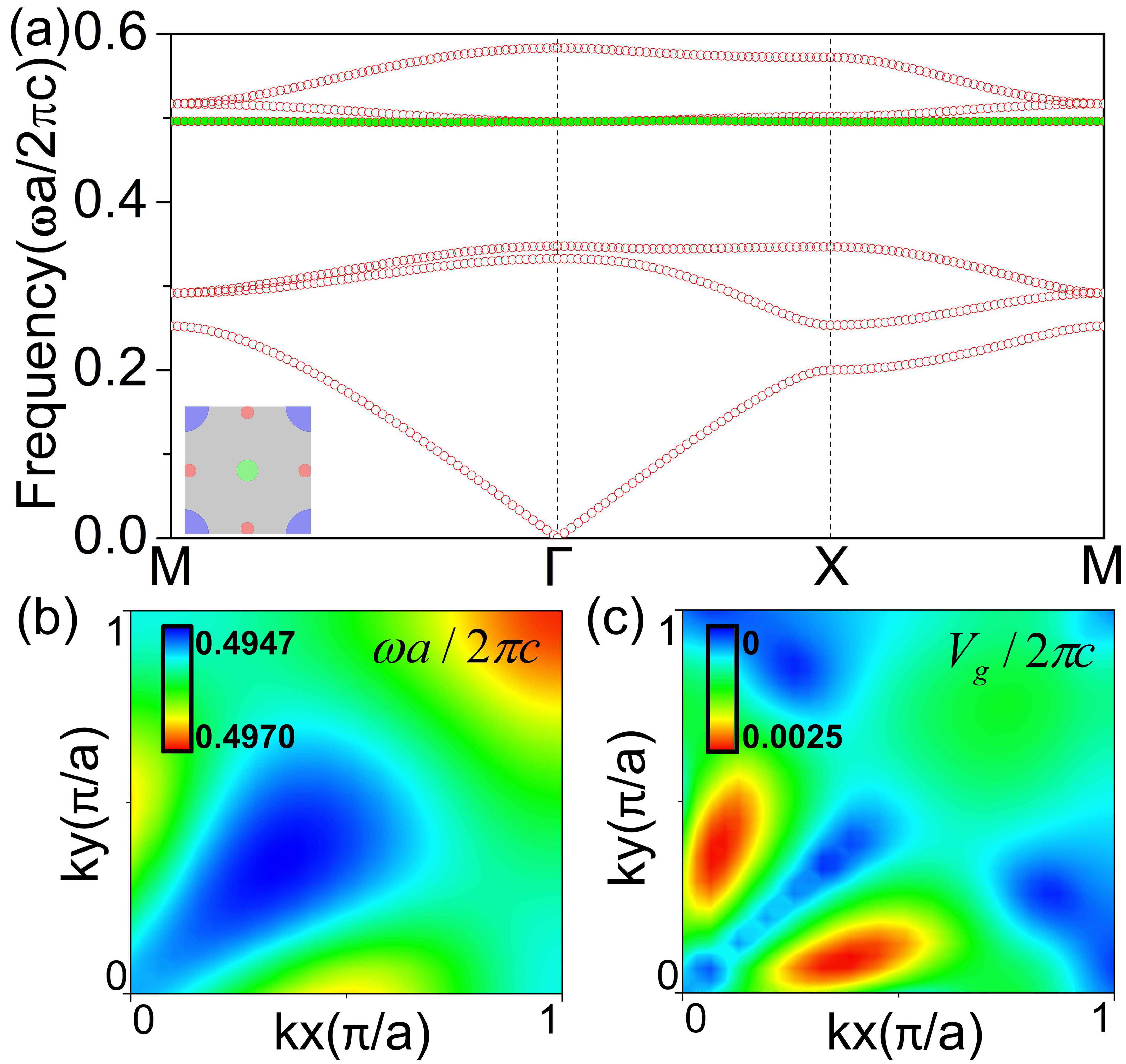


**Fig. S5 | With an additional cylinder 3 at the center of unit cell, the cylinder 2 is set to be tangent to the boundary of unit cell.** (a) The band structure, (b) The EFC, (c) The absolute value of group velocity of the flat band is plotted.
